# Gene-metabolite profile integration to understand the cause of spaceflight induced immunodeficiency

**DOI:** 10.1038/s41526-017-0038-4

**Published:** 2018-01-29

**Authors:** Nabarun Chakraborty, Amrita Cheema, Aarti Gautam, Duncan Donohue, Allison Hoke, Carolynn Conley, Marti Jett, Rasha Hammamieh

**Affiliations:** 1 Integrative Systems Biology, The Geneva Foundation, US Army Center for Environmental Health Research, Frederick, MD 21702-5010 USA; 2 Georgetown University, Georgetown, Washington, DC 20057 USA; 3 Integrative Systems Biology, US Army Center for Environmental Health Research, Frederick, MD 21702-5010 USA; 4 United States Department of Defense Space Test Program, Johnson Space Center, Houston, TX 77058 USA

## Abstract

Spaceflight presents a spectrum of stresses very different from those associated with terrestrial conditions. Our previous study (BMC Genom. **15**: 659, 2014) integrated the expressions of mRNAs, microRNAs, and proteins and results indicated that microgravity induces an immunosuppressive state that can facilitate opportunistic pathogenic attack. However, the existing data are not sufficient for elucidating the molecular drivers of the given immunosuppressed state. To meet this knowledge gap, we focused on the metabolite profile of spaceflown human cells. Independent studies have attributed cellular energy deficiency as a major cause of compromised immunity of the host, and metabolites that are closely associated with energy production could be a robust signature of atypical energy fluctuation. Our protocol involved inoculation of human endothelial cells in cell culture modules in spaceflight and on the ground concurrently. Ten days later, the cells in space and on the ground were exposed to lipopolysaccharide (LPS), a ubiquitous membrane endotoxin of Gram-negative bacteria. Nucleic acids, proteins, and metabolites were collected 4 and 8 h post-LPS exposure. Untargeted profiling of metabolites was followed by targeted identification of amino acids and knowledge integration with gene expression profiles. Consistent with the past reports associating microgravity with increased energy expenditure, we identified several markers linked to energy deficiency, including various amino acids such as tryptophan, creatinine, dopamine, and glycine, and cofactors such as lactate and pyruvate. The present study revealed a molecular architecture linking energy metabolism and immunodeficiency in microgravity. The energy-deficient condition potentially cascaded into dysregulation of protein metabolism and impairment of host immunity. This project is limited by a small sample size. Although a strict statistical screening was carefully implemented, the present results further emphasize the need for additional studies with larger sample sizes. Validating this hypothesis using an in vivo model is essential to extend the knowledge towards identifying markers of diagnostic and therapeutic value.

## Metabolism: cellular energy depletion behind immunodeficiency in space?

Human cells challenged with a bacterial toxin show more signs of energy deficiency when flown in space than when cultured on the ground. Rasha Hammamieh from the US Army Center for Environmental Health Research in Frederick, Maryland, and colleagues exposed human endothelial cells in spaceflight to lipopolysaccharide, an immune response-triggering part of the bacterial membrane. They then collected nucleic acids, proteins and metabolites 4 and 8 h later, and saw a molecular architecture consistent with increased energy expenditure compared to matched control cells grown on Earth. Combined with the researchers’ previous finding that microgravity can induce an immunosuppressive state, the results suggest that energy imbalances potentially lead to problems with protein metabolism that ultimately impair the immune system. The authors propose that reversing this energy depletion could help enhance the immune health of astronauts.

## Introduction

The host immune defense system is vulnerable to spaceflight-induced stress.^1^ Given that microgravity can simultaneously aggravate pathogenicity^2,3^ and suppress host defenses,^1^ we posit that the host–pathogen dynamics assume novel characteristics in space. Comprehensive characterization of host–pathogen relationships is critical in developing mitigation strategies for the many challenges continuously faced by astronauts, such as delayed wound healing^4^ and musculoskeletal atrophy.^5,6^ The immunosuppressed state in spaceflight, resulting in the impaired signaling of cytokines, chemokines, and other growth factors,^1^ potentially plays an essential role in delayed healing.^7^ Prolonged disuse of bone and muscle tissues during space missions promotes bone mineral density loss,^5^ which has been causally associated with key immunological factors.^8–10^ In examining these two space mission-critical health problems concurrently, host immunity emerges as the primary factor that demands special attention. The upcoming deep-space programs reinforce the necessity to unravel the causes of the immunosuppression promoted by microgravity.

To investigate the impact of microgravity on the host–pathogen relationship, we exposed human endothelial cells in vitro to lipopolysaccharide (LPS), a typical outer-membrane endotoxin of Gram-negative bacteria, to elicit an immune response mimicking a bacterial infection.^1^ The transcriptomic results showed significant suppression of genes associated with host defense, suggesting a temporary immunosuppression caused by spaceflight-induced stress. In effect, the host cells failed to adequately respond to LPS during the first 4 h of the LPS stimulation. Our results further suggested that the impaired response to LPS potentially influenced the downstream tumor necrosis factor (TNF) receptor binding, blood cell production, and adaptive immunity. Regulation of microRNA (miRNA) underlies the epigenetic consequences of microgravity on immune health; these epigenetic signatures were associated with oxidative stress and pathogen recognition and became altered in spaceflight.^1^ Multiple indications of recovering immune health were suggested at the transcript level after 8 h of LPS exposure. However, it could be postulated that once host cells were shocked by reduced gravity, they would never be able to fully recover immune functions.^11^ Independent studies of spaceflown cells found residual immunosuppression long after the spaceflight-induced stress was withdrawn.^12,13^

Our previous study took a systems biology approach in profiling miRNAs, mRNAs, and proteins to delineate interactomes; however, this study did not yield insight into the driving factors behind the compromised immunity caused by microgravity^1^. To bridge the knowledge gap, we conducted an untargeted investigation of the global metabolome obtained from the spaceflown endothelial cells. Interrogation of the metabolomics networks could be justified for three primary reasons. First, as the typical end products and key modulators of protein metabolism, the metabolites are directly involved with nutrient consumption and energy production.^14,15^ Therefore, the metabolites play a significant role in host defense and immunoregulation, which typically undertake energy expensive processes.^16^ Secondly, small changes in environmental conditions tend to cause large metabolic changes.^17–19^ Therefore, the metabolites could be used as a robust readout of the spaceflight-induced stress response. Finally, the gene–metabolite cross-talks are pertinent on a longitudinal timescale because there is a significant time lag between the onset of a genomic perturbation and the resulting disturbance in the metabolic landscape.^20–22^ Hence, an in-depth analysis is likely to yield predictive time-critical signatures and pathway-based understanding of the host response to LPS stimulation under spaceflight-induced stress.

Our multivariate network-based approach linked the potential metabolic and transcriptomic panels to mine co-enriched networks. The amino acids, secondary messengers, and cofactors relevant to energy cycles emerged as most likely to be affected. The expression of the amino acid profiles was validated. These validation data were used to mobilize the second round of network analyses to reconstruct the interactions between metabolites and the transcription profile. Our results joined a small but increasing number of studies to characterize complex biological processes using integrated gene–metabolite profiles.^23–27^ Typically, space studies are challenged by small sample size, which is also the key limitation of the present project. To enhance the statistical confidence to the results, we implemented very strict statistical cut-offs and carried out a targeted assay to validate the abundance of amino acids. The final outcome was supported by a recent in vivo study investigating a mouse model launched by STS-135.^27^ Together, the results indicate that reversal of energy depletion in microgravity could be a viable therapeutic strategy to enhance astronauts’ immune health.

## Results

### Comparative differences between transcript and metabolite landscapes

**Fig. 1:**
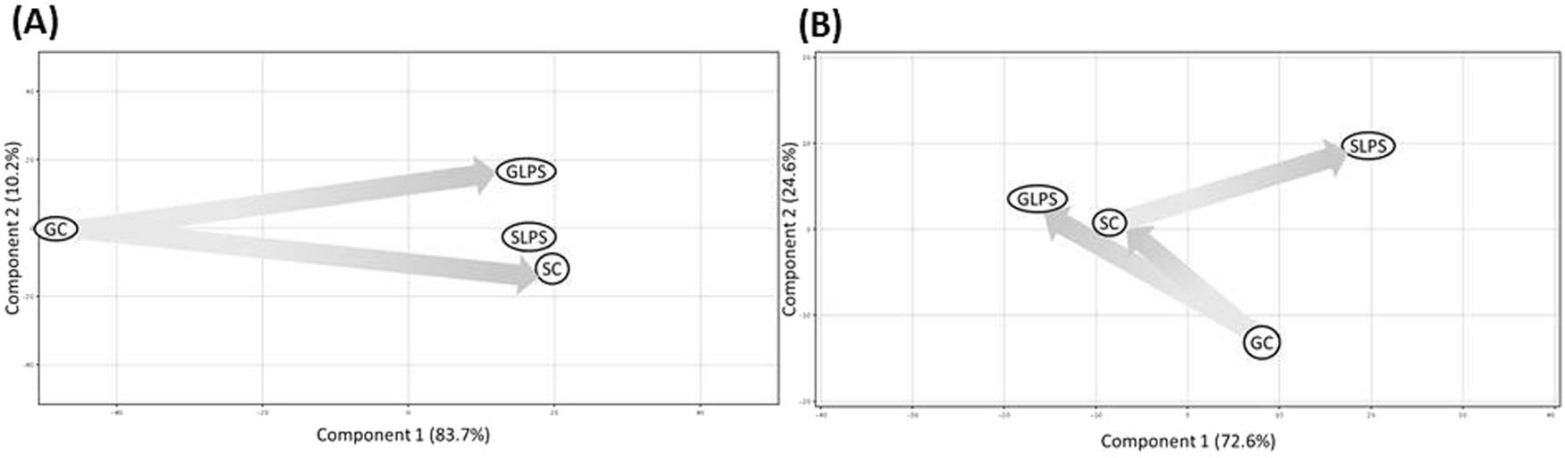
Principal component analysis (PCA). **a** Gene expressions and **b** metabolite loads yielded unambiguous group separation. GC ground control, SC spaceflight control, GLPS 4 h LPS treatment on ground, SLPS, 4 h LPS treatment in spaceflight

Global analyses of gene expression and metabolite abundances showed significant differences caused by spaceflight-induced stress. Gene expression analysis displayed high variability between (i) ground and spaceflight controls and (ii) ground control and 4 h LPS insult on ground (Fig. 1a). Corresponding transcription profiles were clustered far apart across the principal component one (PC1) vs. principal component 2 (PC2), which shared 83.7 and 10.2% of cumulative variance, respectively. A minimum Euclidean distance was noted between the spaceflight control and 4 h LPS insult in space. In contrast, variations in the metabolite loads were different in all three experimental pairs namely (i) ground and spaceflight controls, (ii) ground control and 4 h LPS assault on ground, and (iii) spaceflight control and 4 h LPS assault in space (Fig. 1b), with a cumulative variance in PC1 at 72.6% vs. PC2 at 24.6%.

The variance in global gene and metabolite expressions were further underscored in a two-way ANOVA analysis, where the two driving variables of interest were gravitational changes and LPS assault. Gravitational shift (terrestrial or 1 G vs. microgravity or µG: *p* < 0.001; *F*-value: 92.7) and the interaction between the gravitational shift and LPS assault (µG/1 G × LPS+/−: *p* < 0.001; *F*-value: 60) emerged as significant in driving the variability in the global gene expression landscape. The exclusive impact of LPS (LPS+ vs. LPS−) was insignificant. In contrast, the cumulative effect of all three factors, namely the gravitational shift (1 G vs. µG: *p* < 0.001; *F*-value: 1066.4), LPS insult (LPS+ vs. LPS−: *p* < 0.001; *F*-value: 588.27), and their cross-talk (µG/1 G × LPS+/−: *p* < 0.001; *F*-value: 82.7) emerged as significant in driving the variability of the global metabolite expression landscape.

### Differentially significant transcript and metabolite profiles

We found 2517 genes differentially perturbed by spaceflight-induced stress. Of these, 901 genes were upregulated and 1616 genes were downregulated. The 4 h LPS exposure carried out in spaceflight and on ground resulted in 5379 genes exclusively altered by LPS treatment. In comparison to the ground control, the 4 h LPS treatment on ground upregulated 3031 genes and downregulated 2348 genes. Likewise, the 4 h LPS treatment in spaceflight resulted in overexpression of 2515 genes and underexpression of 2864 genes. All of the gene data were published earlier^1^ and were used for the functional analysis for the present study.

Furthermore, we investigated the metabolite profile of the cell supernatant collected in the sump bags. Using LC-MS analysis ions, we detected significant alterations between terrestrial environment and microgravity with 48 features in ESI+ mode and 242 features in the ESI− mode; a similar analysis found 386 and 99 features (in ESI+ and ESI− modes, respectively) after 4 h LPS treatment that were significantly different between the terrestrial environment and microgravity. Database annotation of these features putatively identified 17 metabolites significantly altered in abundance by extraterrestrial stress and 55 metabolites altered in abundance by the LPS treatment carried out on ground vs. spaceflight. These metabolites were selected for the functional analysis.

### Gene–metabolite functional analysis and assay validation

Gene–metabolite co-enrichment analysis revealed that the majority of the networks were associated with aberrant protein metabolism and relevant deficiencies (Table 1). Therefore, we investigated the amino acids using a targeted mass spectrometry approach. The results (Table 2) showed the loads of seven amino acids were increased and two were decreased by spaceflight-induced stress. The impact of pathogenic assault was calculated by contrasting the LPS treatments carried out in the terrestrial condition vs. microgravity. This revealed that five metabolites were increased and nine were decreased by LPS insult in space. The majority of the metabolites were found to be coordinated with the predicted activities of ATP and energy production, as well as neuroendocrine and musculoskeletal functions.

**Table 1:** The pathways co-enriched by the genes and metabolites regulated by µG

Pathway	# of genes	# of metabolites	*p*-value
Arginine and proline metabolism	10	3	0.001
Urea cycle and metabolism of arginine, proline, glutamate, aspartate, and asparagine	17	3	0.001
Bile acid biosynthesis	3	2	0.009
Arginine:glycine amidinotransferase deficiency (AGAT deficiency)	3	2	0.025
Creatine deficiency; guanidinoacetate methyltransferase deficiency	3	2	0.025
Guanidinoacetate methyltransferase deficiency (GAMT deficiency)	3	2	0.025
Hyperornithinemia with gyrate atrophy (HOGA)	3	2	0.025
Hyperornithinemia hyperammonemia homocitrullinuria [HHH-syndrome]	3	2	0.025
Hyperprolinemia	3	3	0.025

**Table 2:** Characterization of the amino acids identified by targeted screening using the LC/MS platform

Name	Expression caused by:		Relevant to
	µG/1 G	µG + LPS/1 G + LPS	
Ornithine	1.3	−2.9	One of the major precursors of citrulline
Citrulline	2.0	−3.5	- Energy metabolism
			- Inflammation
			- Ammonia excretion
			- NO precursor in endothelial cells
Tryptophan	0.8	−5.0	- Neurological imbalance
			- Serotonin precursor
Phenylalanine	−1.5	4.6	Neurological imbalance
Glycine	1.6	–	Impaired metabolism by scavenging ROS
Creatinine	0.87	–	Impaired metabolism by scavenging ROS
Dopamine	5.6	–	- Immune modulator
			- Energy imbalance
Arginine	–	1.2	- Muscle protein loss
			- Initiator of immune response
			- NO precursor in endothelial cells
Valine	−1.7	–	Collagen or skeletal disorders
Tyrosine	–	4.6	Imbalanced production of neurotransmitters
Carnosine	–	0.9	Impaired metabolism by scavenging ROS
Sarcosine	–	0.54	Vitamin deficiency and hyperinsulinemia
Aspartic acid	–	−10.0	- Impaired metabolism
			- Reduced amino acids and neuroendocrine synthesis
Threonine	–	−2.7	Malabsorption
Glutamine	–	−4.6	Low muscle mass
Histidine	–	−3.7	- Protein-energy wasting
			- Inflammation and oxidative stress
Isoleucine	–	−2.5	Risk of collagen or skeletal disorders
Spermidine	–	−2.7	- Inhibition of translation and cell growth
			- Protects against ROS

Only the significant molecules are listed, and expression values are in Log_1.5_

**Fig. 2:**
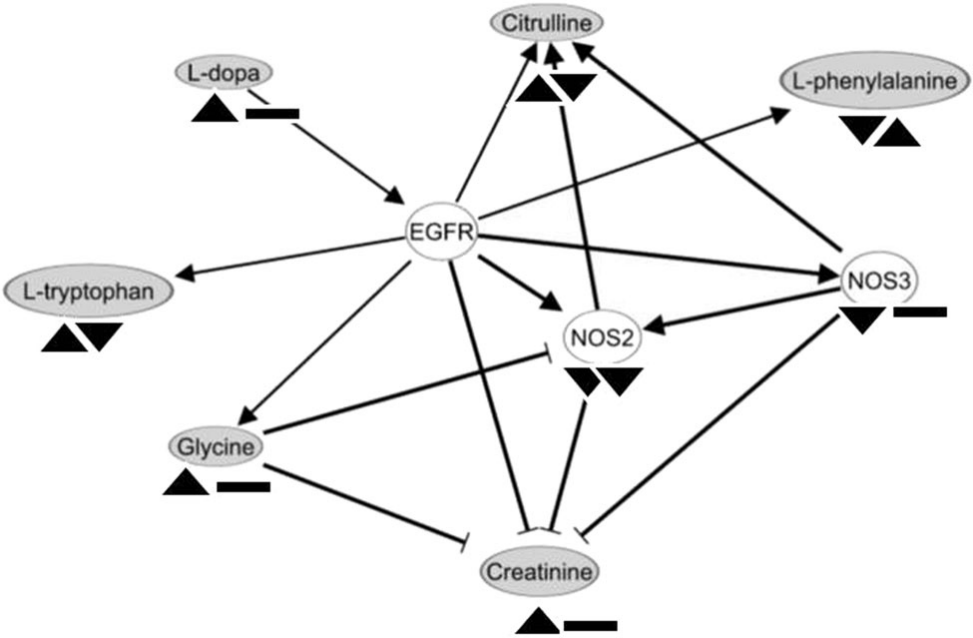
Spaceflight-induced stress in conjunction with LPS insult perturbs energy metabolism. The significantly altered genes and metabolites are integrated to construct this network. The shaded ovals are the metabolites and the edges represent their relationships. The arrowheads under each molecule represent their expressions attributed to microgravity (µG) in comparison to terrestrial condition (1 G) (left), and the 4 h LPS assaults in µG in comparison to 1 G (right). ▲ Represents up, ▼ represents down and ▬ represents no change

Significantly altered networks associated with energy production (Fig. 2) consisted of six metabolites including five increased and one decreased by spaceflight-induced stress. LPS insult reversed the expression pattern of three metabolites in this panel.

**Fig. 3:**
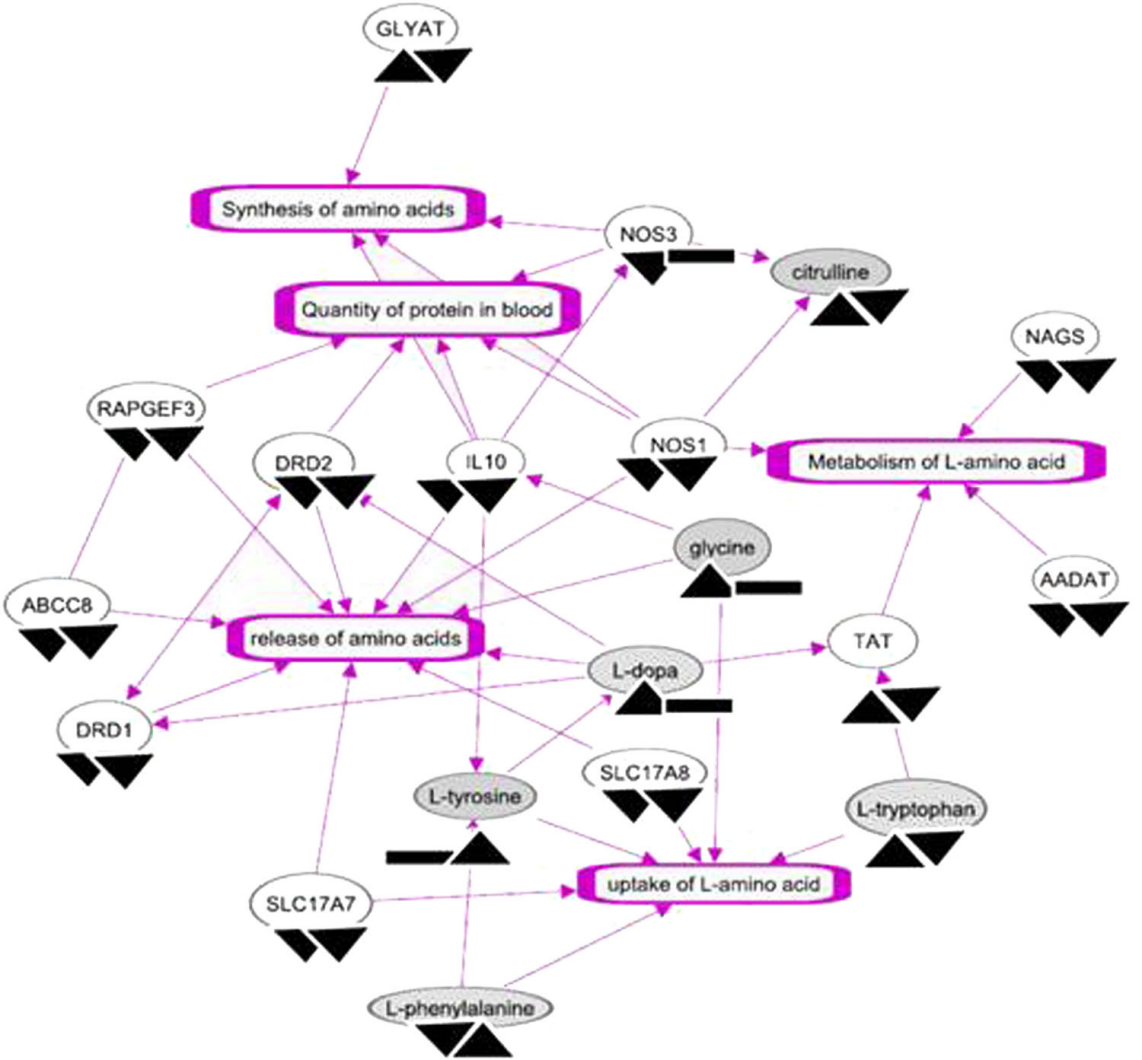
Spaceflight-induced stress in conjunction with LPS insult perturbs amino acid metabolism. The significantly altered genes and metabolites are integrated to construct this network. The shaded ovals are the metabolites and the edges represent their relationships. The arrowheads under each molecule represent their expressions attributed to microgravity (µG) in comparison to terrestrial condition (1 G) (left), and the 4 h LPS assaults in µG in comparison to 1 G (right). ▲ Represents up, ▼ represents down and ▬ represents no change

The amino acid metabolism networks (Fig. 3) were co-enriched by 13 genes and seven metabolites significantly perturbed in spaceflown cells. Five out of seven metabolites associated with amino acid metabolism were upregulated in spaceflight (Table 2). LPS insult reversed the expression patterns of four of these metabolites and two metabolites showed no change upon LPS exposure. Twelve molecules including genes and metabolites of this network were suppressed by spaceflight-induced stress, and LPS assault failed to reverse 11 of these molecules’ expression patterns. More than 60% of these molecules remained underexpressed post-LPS exposure and were specifically involved in the release of amino acids.

The calcium ion network (Figure S1) primarily populated with 40 genes showed comprehensive inhibition as 37 genes were suppressed in spaceflight. LPS assault failed to trigger alteration in the transcriptomic expressions since 30 of these genes remained suppressed after LPS assault in spaceflight.

**Fig. 4:**
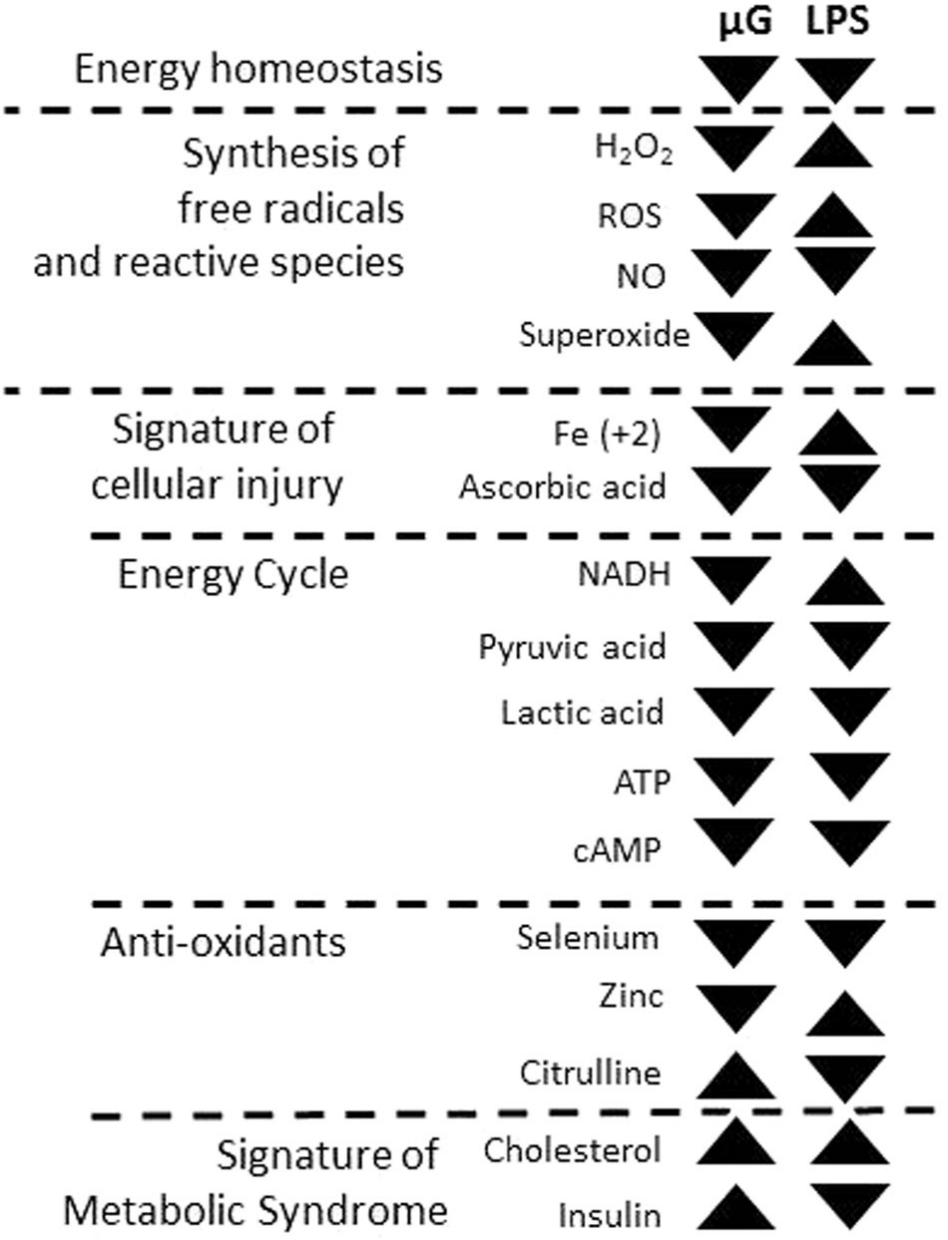
Correlative network analysis indicates predictive activation and inhibition of energy-associated networks caused by spaceflight-induced stress in conjunction with LPS insult: The arrowheads under µG represent their expressions attributed to microgravity (µG) in comparison to terrestrial condition (1 G) (left), and under LPS represents the 4 h LPS assaults in µG in comparison to 1 G (right). ▲ Represents up, ▼ represents down and ▬ represents no change

Functional analysis using the MAP algorithm of Ingenuity Pathway Analysis (IPA) (Fig. 4) predicted that energy homeostasis was suppressed in spaceflight and remained unresponsive to the LPS shock. In contrast, this predictive model indicated that LPS shock potentially reversed the expressions of several energy carriers (such as NADH), reactive species, and free radicals. All of these metabolites were suppressed in microgravity as predicted, but LPS assault stimulated their synthesis in spaceflight. ATP, lactic acid, and pyruvic acid production were inhibited in spaceflight and LPS assault failed to alter their status. The expressions of insulin and cholesterol, two metabolic signatures closely associated with protein metabolism, were overexpressed with spaceflight-induced stress. LPS assault reduced the insulin levels.

## Discussion

The objective of the study was to understand the differential impact of microgravity (µG) in comparison to the typical terrestrial condition (1 G) and to assess the impact of LPS assault during spaceflight (µG + LPS) vs. ground (1 G + LPS). Our past study^1^ suggested that a complex relationship exists between the LPS assault and the microgravity, which often emerged counterintuitive to the inferences obtained from studying the terrestrial condition vs. microgravity. Hence, the present project measured the differences captured in the gene–metabolite axis in microgravity vs. terrestrial condition (µG/1 G). In addition, we investigated the impact of LPS assault on the gene–metabolite axis in microgravity vs. terrestrial condition (µG + LPS/1 G + LPS).

### Microgravity, immunodeficiency, and energy expenditure: a complex nexus

Genes associated with immunological health, and those that were associated with apoptosis and the release of cytokines, chemokines, and growth factors, were suppressed in microgravity.^1^ As a result, the host cells potentially failed to respond 4 h post-LPS challenge. However, after a longer rest period that lasted for 8 h post-LPS assault, more genes began to respond to the LPS stimulation. Taken together, we inferred that spaceflight induced a temporary immunodeficiency, making the host vulnerable for opportunistic infection. Our omics results supported previous reports about the influence of microgravity on the immunological machinery.^13,28–30^ A decreased functionality of immune health and altered corticosterone and lymphocyte abundance in microgravity were observed in both human and rodent cohorts flown into space.^31–34^ Blood samples collected from astronauts showed an overall immunosuppression in response to LPS stimulation in vitro. A host of cytokines, including IL-6, IL-1β, and TNFα, accompanied by phagocytic activity was altered in microgravity.^35,36^

However, the causative factors driving such negative implications of microgravity on immune functions remained elusive. Since a growing number of ground-based studies began linking the energy deficiency to impaired immunological health,^37,38^ we found it logical to explore this subject critically. The gene–metabolite integrative networks indicated an impaired protein metabolism and amino acid deficiency in vitro caused by spaceflight-induced stress. This observation was consistent with previous reports linking the change in gravitational force to the enhanced need for energy.^39^ A recent genomic study of rodent livers found inhibited glycolysis in microgravity^27^ that further supports our findings related to diminishing trends in synthesis of many energy components, such as ATP and cAMP, which potentially contribute to an energy imbalance.

The fluctuating production of energy in the spaceflown cells was highlighted by the accumulation of creatinine,^40^ dopamine,^41^ and glycine.^42^ These molecules are typically recruited to restore the energy balance. Dysregulated energy metabolism is potentially linked to compromised host immunity.^37,38^ The resulting immunodeficiency was highlighted in our previous study that explored the relationships between mRNA, miRNA, and protein. In extending that knowledge, the present investigation of the metabolite landscape revealed an elevated load of tryptophan in microgravity, and it became depleted as a result of LPS assault. Tryptophan deprivation arrested the T-cell cycle, and this ultimately impaired adaptive immunity.^43,44^ Another marker of immunodeficiency was the accumulation of dopamine that promotes neurotoxicity and facilitates opportunistic pathogenic assault by modulating pro-inflammatory signal.^45^ Depletion of valine in spaceflight and the reduced accumulation of isoleucine post-LPS exposure in microgravity were also markers of immunodeficiency.^46^ The LPS assault in spaceflight triggered less accumulation of glutamine that plays an integral role in immunoregulation,^47^ and thereby the LPS response mechanism was further altered.

### Perturbed amino acid profile in spaceflown cells in vitro

Gene–metabolite co-enrichment analysis found significant perturbation of the networks associated with protein metabolism pathways as a consequence of spaceflight-induced stress. Arginine:glycine amidinotransferase (AGAT) activities and creatine metabolism are two closely related networks. Creatine accumulation critically depends on the ornithine transportation across mitochondrial membranes. Together, they control the mitochondrial biogenesis and energy production.^48^ Abnormal accumulation of ornithine, citrulline, and creatine in microgravity possibly initiates a comprehensive dysfunction of mitochondria.^49^ LPS assault decreased the load of ornithine and aspartic acids, which are the markers of hyperammonemia and the potential precursors of acute metabolic decomposition.^50^ Significant loss of these amino acids after LPS stimulation possibly indicates the acute vulnerability of metabolic activities and altered mitochondrial bioenergetics due to the spaceflight-induced stress.

The versatile consequences of compromised mitochondrial bioenergetics are discussed herein primarily in the context of augmented neurodegeneration and impaired calcium buffering.^51–53^ The influence of spaceflight-induced stress on neurodegeneration was marked by significant accumulation of dopamine^54^ and tryptophan^55^ in vitro. The increased load of dopamine potentially resulted in the decline of ATP synthesis and suppressed pyruvate-dependent electron transport, and in turn, possibly inhibited the ATP-coupled respiration.^54^

The intracellular calcium buffering and reactive oxygen species (ROS) production, two major end points of mitochondrial bioenergetics,^53^ were also found perturbed in spaceflight. A comprehensive suppression of ROS synthesis was attributed to restricted mobilization and depleted load of calcium.^56^ A recent study reporting an inhibited synthesis and metabolism of ROS in liver genes of space-flown mice further supported our observation.^27^

Impaired activities of calcium channel synchronized with the dysregulated protein metabolism and energy deficiency could initiate protein metabolism in bone and muscle tissue, and in turn, mediate the musculoskeletal atrophy.^9^ This observation is particularly important since microgravity presents an ideal condition of bone disuse perennially linked to osteoporosis,^6^ although its molecular underpinnings are yet unclear.

More than two-thirds of the molecules enriching the calcium network remained suppressed post-exposure. This observation potentially indicates the compromised host defense and immunosuppression.^56^ The depletion of calcium production during spaceflight was coordinated with suppressed H_2_O_2_ synthesis. Since the calcium surge is directly involved with H_2_O_2_ production at the wound bed,^56^ the present study showed a distinct scenario where LPS assault triggered the production of H_2_O_2_ and superoxide despite the continued depletion of calcium. A rather extreme consequence could be expected in light of depleted spermidine, which enhances protection against ROS-mediated damage.^57^

The surge of ROS and superoxide typically drives recruitment of pro-inflammatory mediators.^58,59^ In spaceflight, the transcriptional signals associated with pro-inflammation became partially overexpressed 8 h post-LPS assault. Corresponding networks and candidate markers could be potential therapeutic targets that could be manipulated to accelerate the inflammatory response.

### Concluding remarks

We previously reported a study that^1^ primarily focused on the functional end points of the hierarchical coordination among miRNA, mRNA, and proteins. The results indicated a temporary immunosuppression due to spaceflight conditions that could facilitate infections by opportunistic pathogens. Integration of metabolomics with transcriptomic-proteomic results was used to mine the molecular markers of energy deficiency in spaceflight and the causal association with immunosuppression and dysregulated calcium buffering. The deficient energy state can initiate catabolism of proteins in muscle and bone tissues as the primary backup source of energy.^12,60^ The present analysis elucidates a potential link between energy deficiency and bone tissue loss, the perennial challenge of space missions.^6^

Limitations of this study include a small sample size; this assay was designed based on the 12 available bioreactors assigned to this project. The compromised statistical power was potentially compensated by implementing cross-omics knowledge integration and platform-specific validation of amino acids. Moderated *t*-test (*p* < 0.1), a preferred tool for screening small sample size^61^ was coupled with a fold change cut-off >|2| to mine the differentially expressed metabolites from the untargeted metabolomics analysis. These differentially expressed metabolites were only used for network building, not for any biomarker discovery. The statistical power of the final outcome was further enhanced by identifying those networks which met the hypergeometric *t*-test *p* < 0.05. Taking cues from the network analysis, a validation study of targeted amino acid discovery was conducted. Furthermore, the present study lacks an in-flight 1xG centrifuge control. Typical hardware constrains and space limitations inside the spaceflight denied the inclusion of this baseline. Moreover, the enrichment analysis presented here was based on the metabolites and chemicals derived from both cellular residues and lysates, and measuring the individual contributions of the time-sensitive sample collections was beyond our scope due to the hardware limitations.

Withstanding these limitations, the present study indicated an important link between immune health and metabolism and suggested their roles in modulating the host health in microgravity. This immunity–metabolism relationship also emerges as one of the principal mediators of obesity and cancer.^62–64^ Reprogramming of metabolism networks was able to modulate the immunity process^65–67^ that, in extension, can potentially regulate the disease pathogenesis.^68,69^ In concurrence, we may postulate that some of the major adversities of astronauts such as bone loss, immunodeficiency, loss of appetite, premature aging, etc., could be ameliorated by strategic manipulation of the immunity–metabolism relationship. Supporting evidence came from recent studies that suggested major roles of immunity and energy/lipid metabolism on spaceflight-induced stress.^27,70,71^ We could further expand our knowledge by following up to target the cell lines with in vitro assays linked to immunity,^72^ kidney,^73^ and liver^71^ functions that are important to spaceflight-induced stress. In fact, our forthcoming study will particularly focus on the role of immunity and metabolism in the potential bone loss in vitro during spaceflight. To address the typical limitations of in vitro outcomes, in vivo studies challenged with specific stress factors could be a valuable addition.^27,71^ Our recently concluded Rodent Research 4 study (https://www.nasa.gov/ames/research/space-biosciences/rodent-research-4-spacex-10) investigated the impact of bone defects during the prolonged skeletal unloading condition in spaceflight. The characterization of the relationship between metabolism and immunity, and their role in tissue healing is underway. Similar studies incorporating additional stress agents such as challenges with relevant toxins could be greatly beneficial. As a long-term goal, we seek the molecular sensors that can potentially predict the discordance between immunity and metabolism, and consequently, predict the onset of specific diseases. In addition, our future goal is to identify the biomarkers that can act as the metabolic switch^74^ to facilitate strategic onset of bioenergetic metabolism essential for survival in the weightlessness condition. Hence, the present outcome could be highly valuable to a next generation therapeutic and rehabilitation program of astronauts.

## Materials and methods

### Ethics statement

The following methods were performed in accordance with relevant regulations and guidelines, and the methods were approved by Walter Reed Army Medical Center, Silver Spring, MD.

### Experimental design

This was an in vitro assay; no live animals were involved. The details of the assay are discussed elsewhere^1^. Briefly, six bioreactors were allotted to us in the STS-135 Atlantis Space Shuttle mission. The bioreactor is a capsule that encloses a bundle of capillary filaments attached to the capsule’s two ends. These filaments transport nutrients and other treatment materials to support the three-dimensional cell inoculation growing in the extra-capillary space of individual filaments. The cell culture module (CCM) (Tissue Genesis, Inc., Honolulu, HI) is a state-of-the-art, feedback controlled, automated platform used to run these bioreactors in the space shuttles and on the ground. The CCM was integrated and flown under the direction of the US Department of Defense (DoD) Space Test Program.

**Fig. 5:**
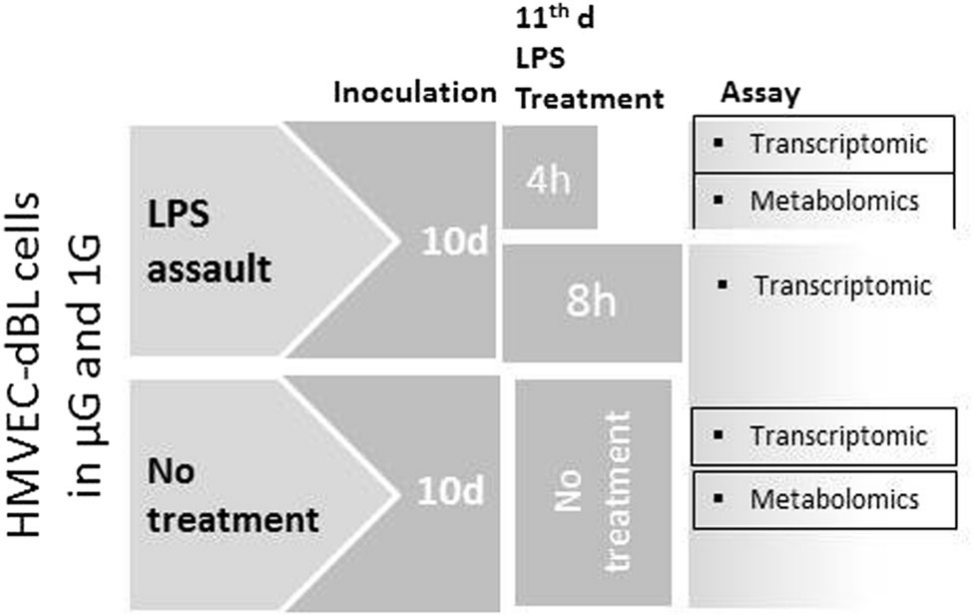
Schematic illustration of the assay conducted simultaneously in spaceflight (STS-135) and on ground. Molecular perturbations caused by LPS assault in both µG and 1 G were assessed in comparison to the no-treatment control in HMVEC-dBL cells. Cell inoculation, treatment, and assay types for two assay variables are noted. The transcriptomic study previously published by us investigated the mRNA–miRNA–protein relationship and how it becomes vulnerable to spaceflight-induced stress. The boxed assay results are used in the present communication

The bioreactors were coated with fibronectin (Sigma Chemical Co., St. Louis, MO) as the cell adhesive agent. One million human dermal blood microvascular endothelial cells (HMVEC-dBL; Lonza, Walkersville, MD) per bioreactor were maintained in EGM-2MV growth media supplemented with growth factors, antimicrobials, cytokines, and 5% fetal bovine serum (all purchased from Lonza). Figure 5 shows an overview of the experimental design and corresponding -omics assays. Briefly, from a group of 12 freshly prepared bioreactors, six were retained on ground and the other six bioreactors were launched to space within 24 h of the cell inoculation. Henceforth, these two batches of six bioreactors each underwent similar assay protocols but were maintained in terrestrial and microgravity, respectively. During the first 10 days of the space expedition, the cell inoculation was supported under optimal conditions. An inbuilt motor facilitated the circulation of cell media from the storage chamber through the bioreactors and finally to storage in the sump bags. On the 11th day, four bioreactors were treated with 100 µg/mL LPS (source was *E*. *coli 055: B5*, Sigma Chemical Co.), two each for 4 and 8 h, respectively. The remaining two bioreactors were left untreated as controls. The experiments were terminated by injecting RLT buffer (QIAGEN, Inc., Gaithersburg, MD) into the bioreactors, which were maintained at room temperature for the rest of the space mission. Upon retrieving the bioreactors on ground, RNA was extracted following a protocol described elsewhere^1^. The sump bags storing the spent media, cellular secretions, and cell lysates, including proteins, metabolites, and chemicals, were also collected; their contents were aliquoted and stored at −80 °C for future assessment.

### Metabolomic analysis

Metabolite profiling was performed in two phases. The untargeted metabolomics analysis was followed by targeted evaluation of the expression of amino acids. In both phases, unused fresh media was loaded as the baseline negative control.

For the untargeted metabolomics assay, each sample (5 μL) was injected onto a reverse-phase 50 × 2.1 mm Acquity 1.7-μm C18 column (Waters Corp, Milford, MA) using an Acquity UPLC system (Waters) with a gradient mobile phase consisting of 2% acetonitrile in water containing 0.1% formic acid (Solvent A) and 2% water in acetonitrile containing 0.1% formic acid (Solvent B) and resolved for 10 min at a flow rate of 0.5 mL/min. The gradient consisted of 100% A for 0.5 min with a ramp of curve 6–100% B from 0.5 to 10 min. The column eluent was introduced directly into the mass spectrometer by electrospray. Mass spectrometry was performed on a Q-TOF Premier mass spectrometer (Waters) operating in either positive-ion or negative-ion electrospray ionization (ESI+/−) mode with a capillary voltage of 3200 V and a sampling cone voltage of 20 V in negative mode and 35 V in positive mode. The desolvation gas flow was set to 800 L/h and the temperature was set to 350 °C. The cone gas flow was 25 L/h, and the source temperature was 120 °C. Accurate mass was maintained by introduction of LockSpray interface of sulfadimethoxine (311.0814 [M+H]+ or 309.0658 [M−H]−) at a concentration of 250 pg/μL in 50% aqueous acetonitrile and a rate of 150 μL/min. Data were acquired in centroid mode from 50 to 850*m*/*z* in MS scanning. Centroided and integrated mass spectrometry data obtained in duplicates from the UPLC-TOFMS were processed using XCMS (Scripps Institute) to generate a data matrix containing ion intensities, mass to charge (*m*/*z*) and retention time values.

For the amino acid targeted assay, internal standards specific for individual amino acids were used. Ten microliters of the internal standard was added to all 1.5 mL Eppendorf vials. With 10 μL of phosphate-buffered saline as the zero sample, calibrant standards and QC solution were then added to the respective vials followed by 20 μL of plasma to the sample vials. The derivatizing reagent was prepared by mixing 950 μL of water, ethanol, and pyridine along with 150 μL of phenyl isothiocyanate. Fifty microliters of this mixture were then added to the vials and left at room temperature for 20 min. The excess liquid was removed from the vials by drying under nitrogen for 60 min, then 300 μL of 5 mM ammonium acetate in methanol was added, and the vials were kept on the shaker for 15 min. The vials were centrifuged at 13,000 rpm for 10 min at 4 °C, and supernatant was transferred to mass spec vials. Five microliters of the samples were injected into an Acquity UPLC- Xevo TQ-S system (Waters). The amino acids were separated on an Acquity UPLC BEH C18 1.7 μm, 2.1 × 50 mm through a mobile phase that consisted of 0.2% v/v of formic acid in both water (A), and acetonitrile (B).The column temperature was maintained at 50 °C with a flow rate of 0.9 mL/min. The gradient began with the polar phase at 100% which was maintained for 0.38 min and gradually decreased to 85% by 3 min and 30% by 5.4 min. The organic phase became 100% by 5.5 min and the initial gradient was reached by 5.93 min which was maintained until 6.6 min. Finally, the data matrix was generated citing the expression levels of individual amino acids.

### Statistical analysis

GeneSpring (Agilent Technologies, Inc., Santa Clara, CA) was used to normalize and analyze the transcriptomic data, as reported elsewhere.^1^ To find significant transcripts, we used a moderated *t*-test with *p* < 0.05.

MS scanning was operated in a negative-ion or positive-ion electrospray ionization mode acquired in centroid mode from 50 to 850*m*/*z*. From the pool of detected ions (signal-to-baseline ratio ≥2), moderated *t*-test (*p* < 0.1) screened positive and negative ions as significantly different between the experimental variables. Using MetaboSearch (http://omics.georgetown.edu/metabosearch.html), 50% of the peaks were annotated by chemical identifiers queried from the four major metabolite databases: Human Metabolome DataBase (HMDB), Madison Metabolomics Consortium Database (MMCD), Metlin, and LipidMaps.

Principal component analysis was used for visualization purposes, and IPA (www.ingenuity.com) was used to identify the gene–metabolite networks. IMPaLA (impala.molgen.mpg.de), a tool dedicated to integrating metabolites and genes, was used to mine networks co-enriched by differentially expressed genes and varying metabolite abundances. Networks showing significant enrichment (*p* < 0.05) were documented in the present project. The Molecular Activity Predictor (MAP) tool provided by IPA was used to predict the functional activities.

### Disclaimers

The views, opinions, and/or findings contained in this report are those of the author and should not be construed as official Department of the Army position, policy, or decision, unless so designated by other official documentation.

Citations of commercial organizations or trade names in this report do not constitute an official Department of the Army endorsement or approval of the products or services of these organizations.

### Data availability statement

We have submitted the microarray data to the Gene Expression Omnibus (GEO): GSE54213.

## Electronic supplementary material

Supplement Figure 1
