# Supplementary figures and images for: Gene-metabolite profile integration to understand the cause of spaceflight induced immunodeficiency
Source: NPJ Microgravity. 2018 Jan 29;4:4. doi: 10.1038/s41526-017-0038-4 (PMC5788863; doi:10.1038/s41526-017-0038-4)

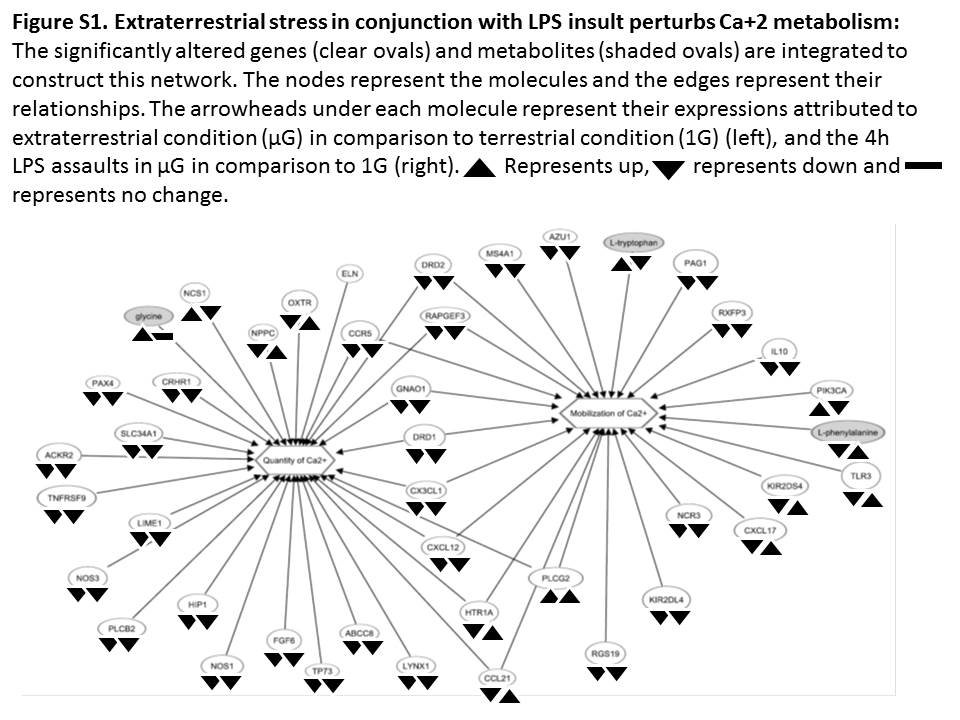

Supplement: Supplementary file 1 — Supplement Figure 1 [file 41526_2017_38_MOESM1_ESM.jpg]
